# Sixth European Hemiptera Congress

**DOI:** 10.3897/zookeys.319.5975

**Published:** 2013-07-30

**Authors:** Alexi Popov, Nikolay Simov, Snejana Grozeva

**Affiliations:** 1 National Museum of Natural History, Bulgarian Academy of Sciences, Tsar Osvoboditel Blvd 1, 1000 Sofia, Bulgaria; 2 Institute of Biodiversity and Ecosystem Research, Bulgarian Academy of Sciences, Tsar Osvoboditel Blvd 1, 1000 Sofia, Bulgaria

Since 1998, when the First European Hemiptera Congress (EHC) took place in the small seaside resort Amaliapolis, Greece, the Hemiptera congresses are regularly held every two-three years: EHC 2 in Fiesa, Slovenia (2001), EHC 3 in St. Petersburg, Russia (2004), EHC 4 in Ivrea, Italy (2007) and EHC 5 in Velence, Hungary (2009).

The nomination to host and organize the Sixth European Hemiptera Congress in Bulgaria, erected during the Fifth Congress in Hungary, had been a surprise and an honour for the Bulgarian hemipterists, and, of course, it is a recognition for the work of Prof. Michail Josifov, renowned taxonomist of Palaearctic Heteroptera, and a tribute to his memory. The geography of the European meetings on Hemiptera shows an interesting trend: five of all six congresses are held on the Balkan Peninsula and the neighbouring countries in south-eastern Europe, Hungary and Italy. The explanation of this fact should be the rich fauna of this region, very suitable for congress trips, more than in many other countries.

Later, the entomologist Alexi Popov, Director of the National Museum of Natural History in Sofia, kindly accepted to be Chairman of the Congress. He is not a hemipterist, but knowing personally some of the remarkable taxonomists in this field, was happy when agree to lead the Organizing Committee and to help the preparation and realizing the Congress.

The institutional co-organizers of the Congress become National Museum of Natural History (Bulgarian Academy of Sciences), Institute of Biodiversity and Ecosystem Research (Bulgarian Academy of Sciences) and St. Kliment Ohridski University of Sofia.

An international recognition of the Congress is the patronage of Director-General of UNESCO Ms Irina Bokova and the promise for financial and other support.

As venue of the Congress, we chose Scaptopara Campus of the American University in Bulgaria, Blagoevgrad. This town, situated 100 km south of Sofia, has a remarkable location. It is situated at the foot of Rila Mts., the highest mountain on the Balkan Peninsula, and is only 30 km from Kresna Gorge in Struma Valley. These are areas with most cold-resistant and most thermophilic fauna in Bulgaria, where many new species of Hemiptera were described from.

The website of the Congress, arranged by the Organizing Committee members and maintained by Ilia Gjonov, comprises the whole information of interest to the participants, including as well the history of the previous congresses. The Organizing Committee published for the participants a booklet by Victor Fet, a well-known taxonomist on scorpions. Prof. Fet (Marshall University, Huntington, West Virginia, USA) for many years combined his biology with literary work involving themes of modern natural science and their philosophy. The booklet consists of three literary pieces: The tale of Prime Minister and a Golden Bedbug (a fairytale on free government elections), The Kirkaldy connection (the generic names used in a novel of Vladimir Nabokov are the generic Hemiptera names of George Willis Kirkaldy) and Zoological label as literary form (also devoted to V. Nabokov).

The Sixth European Hemiptera Congress held from 25 to 29 June 2012 with 100 participants from 26 countries in four continents, including 6 from Bulgaria (Fig. 1). For comparison, the number of participants in each of the previous congresses without these from the host-country is between 30 and 60. More than twice increased number of participants indicates the deep interest to this congress. The significant extension of the geography of European Hemiptera congresses is also impressive. Several countries are presented for the first time in these congresses, among them a large group of hemipterists from countries beyond the border of Europe (17 entomologists from 7 non-European countries or one sixth of participants and more than one fourth of the countries or 27 %). Most hemipterists took part from Poland (15), Czech Republic (9) and Germany, Austria, Hungary, Bulgaria (6 participants each).

**Figure 1.**
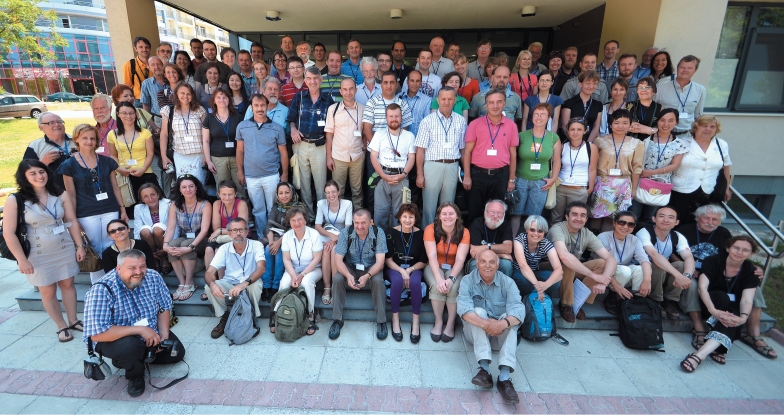
Participants in the Sixth European Hemiptera Congress in front of Scaptopara building, Blagoevgrad, Bulgaria, 26 June 2012. Photo: Werner Holzinger.

During the four days of plenary sessions, 102 reports (45 oral presentations and 57 posters) altogether were delivered and presented, and in this respect the Congress in Bulgaria also exceeds to a large degree the previous ones. There were 11 plenary sessions (Fig. 2), one poster session (Fig. 3) and another session was devoted to the opening ceremony. The short Scratchpads Training course, included in the scientific program of the Congress, is an easy to use, social networking application which enables communities of researchers to manage, share and publish taxonomic data online. It has attracted considerable attention of many participants.

**Figure 2.**
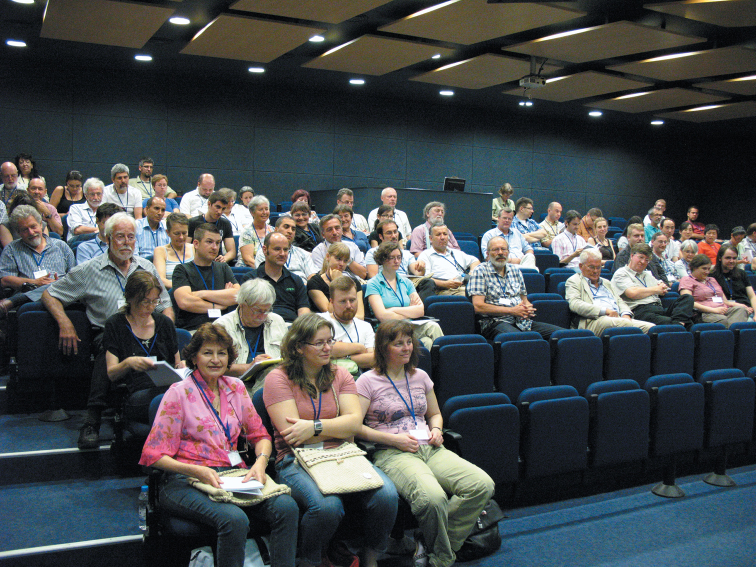
Plenary session in the congress hall, 25 June 2012.

**Figure 3.**
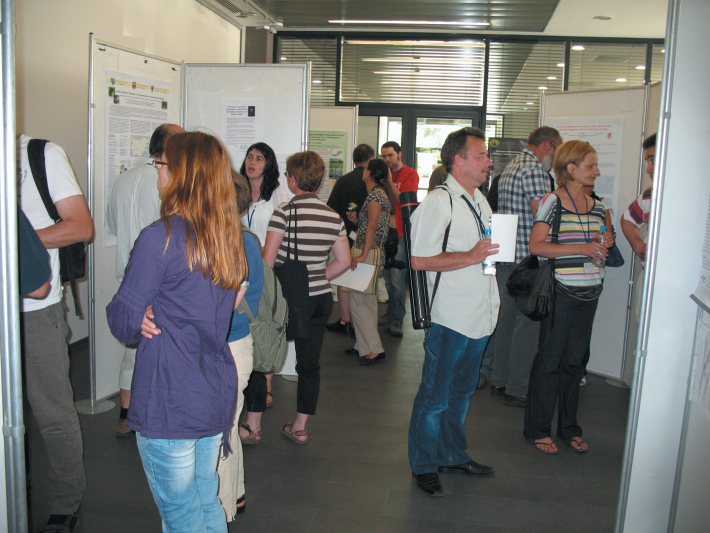
Poster session, 28 June 2012.

The reports presented were focused on general aspects of studies on Hemiptera treating faunistics and biogeography of the Mediterranean Basin and Europe more generally as well as on taxonomy and phylogeny of Cicadomorpha, Fulgoromorpha, Heteroptera, Aphidoidea and Psylloidea; complex application of ecological, acoustic, genetic, palaeontological and behavioural methods; applied research and pest control.

The Congress was opened by the Chairman Assoc. Prof. Alexi Popov and welcome speeches were delivered as well by Prof. Sakis Drosopoulos, initiator of the European congresses and organizer of the First congress; Prof. Matija Gogala, Vice-president of the Slovenian Academy of Sciences and Arts; and Prof. Ernst Heiss, former President of the International Heteropterists Society (Fig. 4).

**Figure 4.**
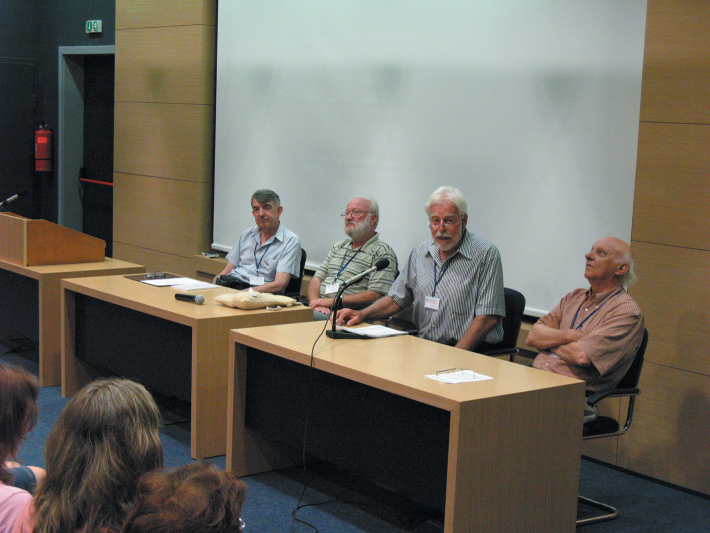
Opening ceremony of the Congress, 25 June 2012, from left to right: Alexi Popov, Matija Gogala, Ernst Heiss, Sakis Drosopoulos.

The Organizing Committee initiated an award for young researchers in memory of Michail Josifov due to the sponsorship of Asen Nikolov Foundation and Pensoft Publishers. The organizers decided to present three equal rewards for achievements in hemipterology and as support of participation in the Sixth Congress. Eleven researchers applied with their abstracts for the award. The Scientific Committee of the Congress discussed the applications and three applicants were chosen as winners by voting: Qiang Xie (China), Ondřej Balvín (Czech Republic) and Vikas Suman (India) (Fig. 5). Certificates and rewards were delivered to the winners by Milena Josifova, the daughter of Michail Josifov.

**Figure 5.**
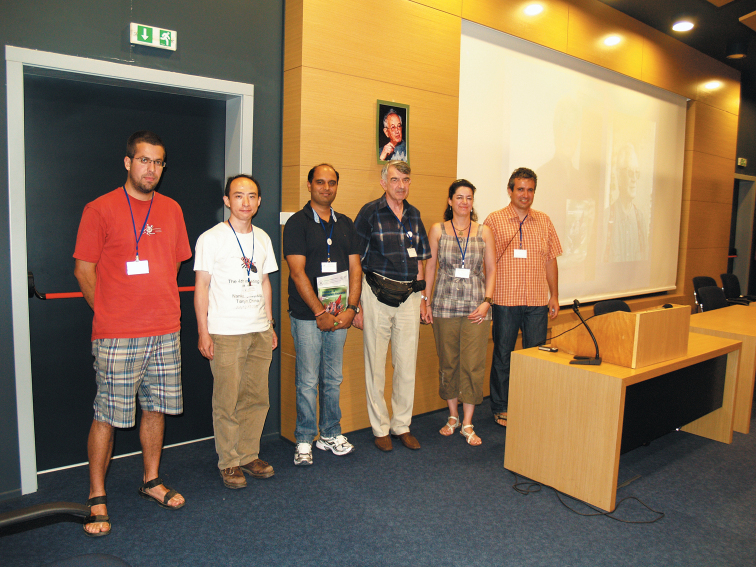
Rewarding ceremony of the winners of Michail Josifov awards, 26 June 2012, from left to right: Ondřej Balvín, Qiang Xie, Vikas Suman (the winners in the competition), Alexi Popov, Milena Josifova, Nikolay Simov. Portrait of Michail Josifov on the wall. Photo: Ilia Gjonov.

First stop of the one-day field trip was Kresna Gorge (Fig. 6). The Kresna Gorge is situated along the Struma River, which passes there between Pirin, Vlahina and Maleshevska mountains. The gorge starts at the Simitli Kettle and ends at the town of Kresna going deep into crystalline schists and granites. Typical habitats for this place are Forests of Grecian juniper (*Juniperus excelsa*); Xerothermic meadows and pastures of *Chrysopogon gryllus*, *Bothriochloa ischaemum* and *Festuca valesiaca*;Sub-Mediterranean pseudo-steppes with annual herbs; Balkan pseudomaquis; Prickly juniper (*Juniperus oxycedrus*) scrub. Part of the territory of the gorge is under the protection of Tisata Reserve and its buffer zone. According to the Bern Convention, it is declared as a CORINE site and will be part of the European Union NATURA 2000 Network. The gorge is of worldwide importance for the conservation of the habitats of the Grecian juniper and the Oriental plane forests. It is also a biological corridor for the migration of large mammals between the surrounding mountain ranges as well as a very important bird migration route (Via Aristotelis). Only among Heteroptera, 419 species are reported from the gorge. Twelve of them are endemic taxa and for nine other the gorge is the type locality. The second collecting place was the area between the Rozhen Monastery and Melnik. The habitats are similar but with forests of *Platanus orientalis* instead of *Juniperus excelsa*, xerothermic meadows and pastures, and sub-Mediterranean pseudo-steppes. Impressive is the picturesque historic town of Melnik. The region comprises the landmark of Melnishki Piramidi, declared as a protected area in 1978 for the purpose of preserving the uniqueness of these earth formations in sand-clay rocks. Because of its European importance for the preservation of rare and threatened habitats, plants and animals, Melnishki Piramidi was declared as a CORINE site in 1998.

**Figure 6.**
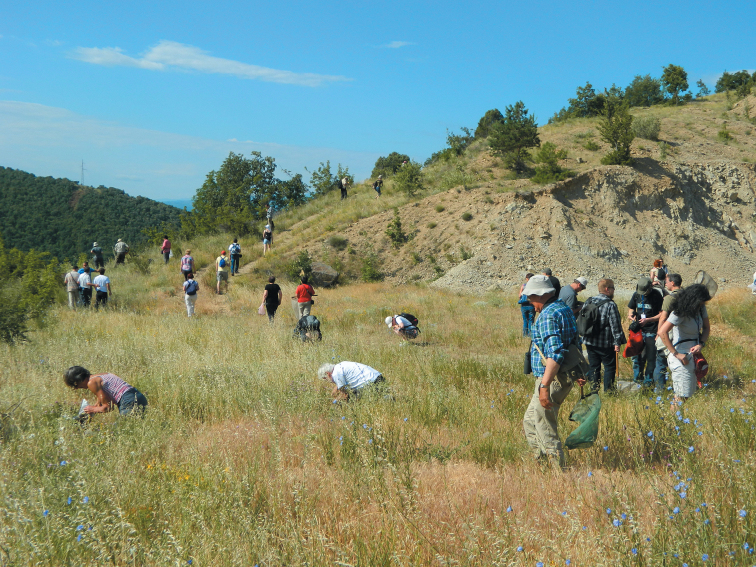
Field trip in Kresna Gorge, 27 June 2012. Photo: Vikas Suman.

Ten participants in the Congress from United Kingdom, the Netherlands, Germany, Austria, Italy, Slovenia and Bulgaria, guided by Ilia Gjonov, took part in an eight-day collecting trip. Localities were visited in Rila Mts. (Rila Monastery), Vlahina Mts., Struma Valley (the hot spring Rupite, the volcanic hill Kozhuh), Pirin Mts. (Popovi Livadi site at 1400 m asl), Western Rhodope Mts. (Shiroka Laka, vicinities of Smolyan, Smolyan Lakes, Snezhanka Peak at 1625 m asl, Sokolovtsi, Popovitsa, Besapara Hills), Eastern Rhodope Mts. (many localities in the vicinities of Momchilgrad, Krumovgrad and Ivailovgrad and along the Byala Reka River) and Sredna Gora Range (Vakarel). The rich collected material of Hemiptera will serve for future investigation of Bulgarian fauna. Various methods for collecting were used, namely hand sampling, sweep netting, light towers, suction samplers. Matija Gogala recorded the songs of some species of Cicadidae. Gernot Kunz photographed many living insects.

The Congress stimulated scientific debates, promoted the communication and cooperation between researchers from different countries, established new contacts and initiated further investigations. We shall keep in our memory the valuable discussions and the exchange of ideas, partly realized and born in the informal meetings during the Welcome party, Congress dinner and Farewell party.

After the congress closing some of the participants visited the National Museum of Natural History in Sofia and its rich collections, especially the valuable collection of Michail Josifov of Palaearctic Heteroptera. At that time, a photo exhibition in the Museum, dedicated to the diversity and significance of Hemiptera for the ecosystems and the people, presented to the audience this significant part of the biodiversity and its conservation. The Congress initiated median interest and public support for the Bulgarian science in these heavy times.

Unfortunately, because of the objective difficulties, UNESCO could not support financially the congress and because of different objective and subjective reasons the publication of this volume was delayed for about half a year.

Thirty-three manuscripts were submitted for the special issue *Advances in Hemipterology*. The selection was made according to the standards of the hosting journal ZooKeys following peer review recommendations and editorial decision. We wish to thank the reviewers for the valuable and creative remarks during the selection process and the improvement of the manuscripts accepted for this volume, which are the first successfully published proceedings of the European Hemiptera congresses.

## Scientific Committee

Berend Aukema (The Netherlands)

Thierry Bourgoin (France)

Ping-ping Chen (The Netherlands)

Sakis Drosopoulos (Greece)

Hans Duffels (The Netherlands)

Alexandr Emeljanov (Russia)

Vladimir Gnezdilov (Russia)

Matija Gogala (Slovenia)

Ernst Heiss (Austria)

Valentina Kuznetsova (Russia)

Pavel Lauterer (Czech Republic)

Rimantas Rakauskas (Lithuania)

Pavel Štys (Czech Republic)

Michael Wilson (United Kingdom)

## Organizing Committee

Alexi Popov (Chairman)

Rumiana Decheva

Ilia Gjonov

Snejana Grozeva

Nikolay Simov

Elena Tasheva

## Participants

Biljana Atanasova (Macedonia)

Berend Aukema (The Netherlands)

Ondřej Balvín (Czech Republic)

Petr Baňař (Czech Republic)

Kees den Bieman (The Netherlands)

Dimitri Boissonnis (France)

Thierry Bourgoin (France)

Milena Březíková (Czech Republic)

Christoph Bückle (Germany)

Attilio Carapezza (Italy)

Ping-ping Chen (The Netherlands)

Leonidas-Romanos Davranoglou (Greece)

Sakis Drosopoulos (Greece)

Johanna Drosopoulou (Greece)

Hans Duffels (The Netherlands)

Ahmet Dursun (Turkey)

Alice Exnerová (Czech Republic)

Zhonghua Fan (China)

Franco Faraci (Italy)

Meral Fent (Turkey)

Kinga Fetykó (Hungary)

Brenda Fürstenau (Brazil)

Beata Gabryś (Poland)

Christian Gissing (Austria)

Ilia Gjonov (Bulgaria)

Matija Gogala (Slovenia)

Gazi Görür (Turkey)

Marta Goula (Spain)

Maria Grandova (Ukraine)

Jocelia Grazia (Brazil)

Snejana Grozeva (Bulgaria)

Susanne Grube (Germany)

Adalgisa Guglielmino (Italy)

Viktor Hartung (Germany)

Masami Hayashi (Japan)

Ernst Heiss (Austria)

Werner Holzinger (Austria)

Dušanka Jerinić-Prodanović (Serbia)

Łukasz Junkiert (Poland)

Małgorzata Kalandyk-Kołodziejczyk (Poland)

Balázs Kiss (Hungary)

Petr Kment (Czech Republic)

Elöd Kondorosy (Hungary)

Gernot Kunz (Austria)

Valentina Kuznetsova (Russia)

Pavel Lauterer (Czech Republic)

Teresa Leszczyńska (Poland)

Bogumił Leszczyński (Poland)

Agata Lis (Poland)

Laurence Livermore (United Kingdom)

Marchal Lorène (France)

Laamari Malik (Algeria)

Anna Maryańska-Nadachowska (Poland)

Peter John Mazzoglio (Italy)

Fariba Mozaffarian (Iran)

Roland Mühlethaler (Germany)

Krzysztof Musik (Poland)

Adam Nadachowski (Poland)

Herbert Nickel (Germany)

Nico Nieser (The Netherlands)

David Ouvrard (United Kingdom)

İnanç Özgen (Turkey)

Diana Percy (United Kingdom)

Olivera Petrović-Obradović (Serbia)

Sophie Pointeau (France)

Alexi Popov (Bulgaria)

Yuri Popov (Russia)

Rimantas Rakauskas (Lithuania)

Alex Ramsay (United Kingdom)

Dávid Rédei (Hungary)

David Sadílek (Czech Republic)

Lydia Schlosser (Austria)

Gabrijel Seljak (Slovenia)

Cezary Sempruch (Poland)

Cecilia Şerban (Romania)

Ewa Simon (Poland)

Nikolay Simov (Bulgaria)

Adeline Soulier-Perkins (France)

Dušan Spasov (Macedonia)

Adam Stroiński (Poland)

Pavel Štys (Czech Republic)

Vikas Suman (India)

Dariusz Swierczewski (Poland)

Éva Szita (Hungary)

Jacek Szwedo (Poland)

Elena Tasheva (Bulgaria)

Attila Torma (Hungary)

Katinka Varga (Hungary)

Jitka Vilimova (Czech Republic)

Meta Virant-Doberlet (Slovenia)

Andja Vučetić (Serbia)

Rong-rong Wang (China)

Yanhui Wang (China)

Michael Wilson (United Kingdom)

Qiang Xie (China)

Mariya Yovkova (Bulgaria)

Selcuk Yurtsever (Turkey)

Ünal Zeybekoğlu (Turkey)

Mathias Zilch (Germany)

Agnieszka Zwolińska (Poland)

